# A modified vaccinia Ankara vaccine vector expressing a mosaic H5 hemagglutinin reduces viral shedding in rhesus macaques

**DOI:** 10.1371/journal.pone.0181738

**Published:** 2017-08-03

**Authors:** Kelsey R. Florek, Attapon Kamlangdee, James P. Mutschler, Brock Kingstad-Bakke, Nancy Schultz-Darken, Karl W. Broman, Jorge E. Osorio, Thomas C. Friedrich

**Affiliations:** 1 Department of Pathobiological Sciences, University of Wisconsin-Madison, School of Veterinary Medicine, Madison, Wisconsin, United States of America; 2 Department of Veterinary Medicine, Kasetsart University, Bangkok, Thailand; 3 Wisconsin National Primate Research Center, Madison, Wisconsin, United States of America; 4 Department of Biostatistics and Medical Informatics, University of Wisconsin School of Medicine and Public Health, Madison, Wisconsin, United States of America; Deutsches Primatenzentrum GmbH - Leibniz-Institut fur Primatenforschung, GERMANY

## Abstract

The rapid antigenic evolution of influenza viruses requires frequent vaccine reformulations. Due to the economic burden of continuous vaccine reformulation and the threat of new pandemics, there is intense interest in developing vaccines capable of eliciting broadly cross-reactive immunity to influenza viruses. We recently constructed a “mosaic” hemagglutinin (HA) based on subtype 5 HA (H5) and designed to stimulate cellular and humoral immunity to multiple influenza virus subtypes. Modified vaccinia Ankara (MVA) expressing this H5 mosaic (MVA-H5M) protected mice against multiple homosubtypic H5N1 strains and a heterosubtypic H1N1 virus. To assess its potential as a human vaccine we evaluated the ability of MVA-H5M to provide heterosubtypic immunity to influenza viruses in a non-human primate model. Rhesus macaques received an initial dose of either MVA-H5M or plasmid DNA encoding H5M, followed by a boost of MVA-H5M, and then were challenged, together with naïve controls, with the heterosubtypic virus A/California/04/2009 (H1N1pdm). Macaques receiving either vaccine regimen cleared H1N1pdm challenge faster than naïve controls. Vaccination with H5M elicited antibodies that bound H1N1pdm HA, but did not neutralize the H1N1pdm challenge virus. Plasma from vaccinated macaques activated NK cells in the presence of H1N1pdm HA, suggesting that vaccination elicited cross-reactive antibodies capable of mediating antibody-dependent cell-mediated cytotoxicity (ADCC). Although HA-specific T cell responses to the MVA-H5M vaccine were weak, responses after challenge were stronger in vaccinated macaques than in control animals. Together these data suggest that mosaic HA antigens may provide a means for inducing broadly cross-reactive immunity to influenza viruses.

## Introduction

Influenza viruses circulate globally, resulting in 3 to 5 million cases of influenza illness each year [1]. Current vaccines can prevent influenza disease, but their effectiveness is highly dependent on the antigenic match between vaccine strains and circulating strains [1–3]. Influenza viruses gradually accumulate mutations that alter antibody recognition. Because of this “antigenic drift,” influenza vaccines must be frequently updated to ensure they match the antigenic properties of circulating viruses [4–6]. Seasonal influenza vaccines are designed to stimulate neutralizing antibodies and are solely evaluated by their ability to elicit antibodies capable of disrupting sialic acid receptor binding, defined as a serum titer of at least 1:40 in a hemagglutination-inhibition (HI) assay [7,8]. A large majority of these neutralizing antibodies are specific for the structurally variable globular head domain of HA [9].

In 2013 the World Health Organization set a goal to have a “universal” influenza vaccine in a phase III efficacy trial by 2020 [10]. A major approach to developing such vaccines has been to elicit antibodies against the conserved stem region of HA [11–13]. However, this has proven difficult due to the immunodominance of antibodies against the globular head [14]. Several strategies have successfully stimulated HA stem-specific antibodies in mice capable of neutralizing a broad range of influenza virus strains in vitro, but few studies have employed these strategies in a translatable model [6,12,13]. Interestingly, Fc-FcγR interactions appear to be necessary for protection mediated by both neutralizing and non-neutralizing antibodies *in vivo* using the murine model [15–18]. This suggests that Fc-mediated effector functions, including antibody-dependent cell-mediated cytotoxicity (ADCC), may play a more important role in broad protection against influenza viruses than previously recognized. In our previous study we showed that vaccination of rhesus macaques with a modified vaccinia Ankara (MVA) vector expressing H5N1 HA elicited “ADCC antibodies,” which were associated with reduced viral shedding upon challenge with the heterosubtypic virus A/Norway/3478/2009 (H1N1); there was not strong evidence for the involvement of cross-reactive T cells in this partial protection [19]. Here we have chosen to use a different H5 antigen to further examine the potential for a vaccine capable of stimulating broadly cross-reactive immunity to influenza.

Mosaic vaccine antigens are designed to represent diverse viral populations while retaining the structural properties of natural proteins. Mosaic HIV antigens have been used to increase the epitopic breadth of T cell responses to vaccination [20]. To extend this approach to influenza vaccines, we engineered a mosaic H5 HA delivered by an MVA vector. This vaccine protected mice against lethal challenge with multiple H5N1 virus clades and the H1N1 virus A/Puerto Rico/8/1934, but not H3N2 viruses; this protection was associated with cross-reactive CD8^+^ T cell responses [21,22]. Thus we reasoned that the mosaic HA construct might more effectively stimulate cross-reactive T cells than our previous construct, which used a wild type HA. To estimate the translatability of this approach to humans, we evaluated heterosubtypic protection in non-human primates, which are more representative of human physiology and immune responses [23–25]. The mosaic H5 antigen was administered to rhesus macaques in either a homologous prime-boost regimen using 2 doses of MVA-HA5, or in a heterologous regimen with a DNA prime and MVA boost. MVA can be potently immunogenic when administered in a heterologous prime/boost strategy [26,27]. We specifically choose a DNA-vectored heterologous prime as DNA is a safe and relatively stable vaccine modality that can stimulate T cell immunity and could be translated to clinical use [28].

The vaccinated animals, together with naïve controls, were then challenged with the heterosubtypic 2009 pandemic virus A/California/04/2009 (H1N1pdm). We evaluated cellular and humoral immune responses to vaccination and challenge, and assessed virus replication in the upper and lower respiratory tracts. Homologous and heterologous prime-boost regimens elicited similar levels of antibodies capable of binding HA, and activating NK cells in the presence of H1N1pdm HA. However, neither regimen elicited potent HA-specific T cell responses prior to challenge, although modest responses became detectable by interferon (IFN)-γ Elispot after challenge. Vaccinated animals cleared H1N1pdm infection more rapidly than control animals. Taken together these findings suggest that MVA-H5 mosaic vaccination can elicit cross-reactive, non-sterilizing protection against influenza viruses, which may be mediated by ADCC antibodies.

## Materials and methods

### Cells and viruses

Chick embryo fibroblasts (CEF) and Madin-Darby canine kidney epithelial (MDCK) cells were obtained from Charles River Laboratories, Inc. (Wilmington, WA, USA), and the American Type Culture Collection (ATCC, Manassass, VA, USA), respectively. Cells were cultured in Dulbecco’s modified Eagle’s medium (DMEM) (HyClone, Logan, UT, USA) supplemented with 10% fetal bovine serum (FBS) (Corning Life Sciences, Corning, NY) and antibiotics. CEFs were used for propagating MVA. Highly pathogenic avian influenza (HPAI) H5N1 virus A/Vietnam/1203/04 (clade 1) was kindly provided by Yoshihiro Kawaoka (University of Wisconsin-Madison, Madison, WI, USA). All experimental studies with highly pathogenic avian influenza H5N1 viruses were conducted in a biosafety level 3+ (BSL3+) facilities in compliance with the University of Wisconsin—Madison Office of Biological Safety. Pandemic H1N1 seasonal influenza virus A/California/04/2009 was kindly provided by Stacey Schultz-Cherry (St. Jude Children’s Research Hospital, Memphis, TN, USA). All viruses were propagated and titrated in MDCK cells with DMEM (HyClone, Logan, UT, USA) that contained 1% bovine serum albumin and 20 mM HEPES (Gibco by Thermo Fisher Scientific, Waltham, MA USA), and 2 μg/ml of TPCK-treated trypsin was added for H1N1 viruses. Viruses were stored at -80°C until use. Viral titers were determined by standard plaque assay using MDCK cells and expressed as pfu/ml.

### Animals

Sixteen male and female (8 males; 8 females) rhesus macaques (*Macaca mulatta*) between the ages of 7 and 15 years old (average age of 10.4 years) were used in this study. One female macaque was dropped from the study, due to preexisting influenza antibody titers. Additionally, r03087, r03089, r03137, r04077, r05092, and r04052 were used from our previous study as historical controls for viral loads; these animals were infected with the same virus stock and dose, and via the same route, as animals in the present study [29].

### Ethics statement

The study was conducted according to the guidelines of the United States National Research Council [30] and the Weatherall Report [31] under a protocol approved by the University of Wisconsin Graduate School Animal Care and Use Committee (IACUC protocol #G00747). Non-human primates were individually housed during the study with visual and auditory access to conspecifics. Standard chow with fruit or vegetables was provided daily with foraging enrichment devices at least weekly. All animals were observed at least twice daily for health or welfare issues. Blood draws were performed under ketamine sedation while other procedures (virus inoculations and brochoalveolar lavages) were performed under ketamine/medetomidine anesthesia and all efforts were made to minimize suffering.

### MVA and DNA vaccine construction

MVA expressing mosaic H5 hemagglutinin (MVA-H5M) was previously constructed as described [21]. Briefly, a mosaic H5 protein was generated *in silico* from the Mosaic Vaccine Designer tool (http://www.hiv.lanl.gov/content/sequence/MOSAIC/makeVaccine.html) with 2,145 H5N1 HA input sequences that included all H5N1 clades. Sequences were obtained from National Center for Biotechnology Information (NCBI), excluding incomplete and redundant sequences. The epitope length was set to 12 to optimize for the length of T helper cell epitopes. The mosaic H5 was reverse-translated into DNA and codon-optimized using GenScript custom gene synthesis (Genscript, NJ, USA). The mosaic H5 DNA was introduced into an MVA under the control of the SE/L promoter to generate MVA expressing mosaic H5 hemagglutinin (MVA-H5M) [32]. The MVA-H5M stock virus was selected and amplified in CEF cells.

We also constructed a DNA plasmid expressing the mosaic H5 hemagglutinin gene (pCMV-H5M). Briefly, the mosaic H5 DNA sequence used in the MVA-H5M construct was cloned into a pCMV vector under CMV promoter. Plasmid preparations for vaccine delivery were confirmed by Sanger DNA sequencing. Then plasmid DNA was precipitated onto 1.6 μm gold particles at a rate of 2 μg DNA per mg of gold for use in the Helio gene-gun vaccination system (BioRad, Hercules, USA). Each gold bullet contained 1 μg of plasmid DNA.

### Vaccination and viral challenge

Fifteen influenza-negative rhesus macaques were divided into 3 groups according to vaccine regimen. Six macaques were primed with MVA-H5M vaccine (1x10^8^ pfu/dose) delivered intradermally, and boosted with MVA-H5M two weeks later. The other seven macaques were primed with 16 μg DNA vaccine delivered transdermally using the Helio gene-gun system (BioRad, Hercules, USA) with 300 psi helium pressure, then 2 weeks later was boosted with MVA-H5M (1x10^8^ pfu/dose). Two macaques (male and female) were used as an unvaccinated negative control. Analyses reported here also include 6 historical controls that were inoculated with same dose of the same virus stock using the same route in a previous study [29].

Eight weeks after the boost, animals were challenged using the same protocol and viral stocks as a previous study, with 9x10^6^ pfu of influenza virus A/California/04/2009 (H1N1pdm) [29]. The total viral inoculum was divided and administered to the trachea, tonsils and conjunctivae as described previously [29]. Virus replication in the upper and lower respiratory tracts was monitored using standard plaque assays on nasal wash and bronchoalveolar lavage (BAL) samples. Weight loss, clinical signs and health status were monitored for one month.

### Enzyme-linked immunosorbent assay (ELISA) for detecting HA-binding antibodies

The ELISA assay was used to detect binding antibodies against HA protein. Briefly, purified HA from influenza virus A/Vietnam/1203/04 (BEI Resources NR-10510) or influenza virus A/California/04/2009 (H1N1pdm) (BEI Resources NR-13691) was coated into Costar™ 96-Well EIA/RIA Plates (Corning Life Sciences, Corning, NY) at 200 ng/ml concentration in PBS, then incubated 4°C overnight. Plates were then washed 3 times in PBS-0.1% Tween-20, and 100 μl of 5% skim milk + 2% FBS (Corning Life Sciences, Corning, NY) in PBS-0.1% Tween 20 was added to each well to blocking non-specific binding. Plates were incubated for 1 hour at ambient temperature, and diluted NHP plasma samples were then added into each well in duplicate and incubated for 1 additional hour at ambient temperature. Plates were then washed 3 times with PBS-0.1% Tween-20. Mouse anti-human IgG antibody clone G18-145 conjugated with horseradish peroxidase (BD Biosciences, San Jose, CA) at 1:1000 dilution was then added into each well and incubated for 1 hour at ambient temperature, followed by 3 washes with PBS containing 0.1% Tween 20. TMB substrate solution (Sigma-Aldrich, St. Louis, MO) was added, and incubated for 20 minutes until blue color was observed, at which time the reaction was stopped and the absorbance at 450 nm of each well was measured with a microtiter plate reader (BLx800 BioTek, Winooski, VT). The limit of detection was at a dilution of 1:15 for A/California/04/2009 (H1N1pdm), 1:50 for A/Vietnam/1203/04, or 1:100 for post-challenge.

### Hemagglutination inhibition assay

The hemagglutination inhibition (HI) assay is used as a standard for assessing the development of vaccine-induced antibodies. This assay was performed according to the protocol described by the World Health Organization [33]. Briefly, 1 part plasma was mixed with 3 parts receptor-destroying enzyme (RDE, Denka Seiken, Tokyo, Japan) and incubated at 37°C for 16 hr to remove non-specific inhibitors of hemagglutination. RDE was then inactivated by incubating the samples at 56°C for 30 min. The 1:4 diluted plasma was then diluted further in serial two-fold dilutions and mixed with 4 HA units of either H1N1 (live A/California/04/2009) or H5N1 (inactivated A/Vietnam/1203/2004) viruses in a U-bottomed microtiter plate. The plates were incubated at room temperature for 30 min following which 0.5% chicken red blood cells (Poultry Sciences University of Wisconsin–Madison, Madison, WI) were added. The samples were incubated at room temperature for 30 min. The reciprocal of the dilution at which no inhibition was observed was recorded as the HI titer. Wells with 4 HA units of virus and PBS were kept as positive and negative controls for hemagglutination. The limit of detection was at a 1:8 dilution; if hemagglutination was observed at a dilution of 1:8, the HI titer was reported as 4 for data analysis.

### Plaque reduction neutralization test

We performed the plaque reduction neutralization test as described previously [34]. Briefly, we used protein G HP SpinTrap columns (GE Healthcare Life Sciences, Pittsburgh, PA) to purify total IgG from plasma obtained from rhesus macaques. We followed the manufacturer’s protocol, except that plasma was incubated with the protein G Sepharose for 1 hour with mild shaking, instead of the standard 5 minutes. Eluted IgG was buffer exchanged in PBS by using an Amicon Ultracel- 30K centrifugal filter unit (Millipore, Billerica, MA) with a 30-kDa molecular mass cutoff in a swinging bucket rotor. Protein concentrations of IgG were measured using the Quick Start^TM^ Bradford Protein Assay (Bio-Rad Laboratories, Inc., Hercules, CA). A/California/04/2009 (H1N1pdm) stock virus was diluted to approximately 50 PFU/well and incubated with 3-fold serial dilutions of total IgG (150μg/ml, 50 μg/ml, 5.5 μg/ml, 1.8 μg/ml) for 1 hour at room temperature. Twelve-well plates were seeded with MDCK cells and washed twice with PBS. Then, 300 μL antibody-virus mixture was placed in duplicate over the MDCK monolayer for 45 minutes at 37°C. The antibody-virus mixture was then aspirated off and the monolayer was washed once with PBS. One ml agar overlay with TPCK treated trypsin and an IgG concentration matching the IgG concentration already present, were added to each well. Plates were incubated for 2 days at 37°C and then fixed with 10% formalin at room temperature for 1 hour. Plaques were visualized and counted by eye. Positive control wells contained no antibody and negative control wells contained no virus or antibody. The limit of detection was set by the dilution limits 150 μg/ml and 1.8 μg/ml.

### Antibody-dependent cell cytotoxicity (ADCC) assay

We measured the ability of plasma antibody to activate NK cells in the presence of HA proteins using a modification on a protocol described previously [35]. Briefly, 96-well plates were coated overnight with recombinant A/California/04/2009 (H1N1pdm) HA proteins expressed from mammalian cells (Sinobiologicals, Shanghai, China). Wells were then washed multiple times with phosphate-buffered saline (PBS) to remove unbound proteins. Heat-inactivated (56°C for 1 hour) EDTA-anticoagulated macaque plasma diluted 1:10 in PBS was then added to each well and incubated at 37˚ C for 2 hours. Wells were again washed repeatedly with PBS. Next, 2x10^5^ NK cells expressing CD16 (KHYG-1) kindly provided by David Evans (University of Wisconsin-Madison, Madison, WI, USA) were added to each well in complete RPMI medium containing 10% fetal calf serum (HyClone, Logan, UT), together with anti-human-CD107a-APC-H7 (H4A3 clone, BD Biosciences, San Jose, CA), 5 μg/ml Brefeldin A (Sigma, St. Louis, MO) and 5 μg/ml Monensin (Golgi Stop, BD Biosciences, San Jose, CA) [36]. Plates were incubated for 5 hours at 37°C, after which time cells were incubated with the following antibodies for 30 min at room temperature: anti-CD16 (clone 3G8, BD Biosciences, San Jose, CA) anti-NKG2A PC7 (clone Z199, Beckman Coulter, Brea, CA) and Live Dead Stain Near IR (Thermo Fisher Scientific, Grand Island, NY). Finally, cells were fixed with 1% paraformaldehyde and acquired on an LSRII flow cytometer (BD Biosciences). Data were analyzed using FlowJo Version 10.0.

### IFN-γ ELISPOT for detecting antigen specific T cells

PBMC were separated from whole EDTA-treated blood by Ficoll-Paque PLUS (GE Health Sciences) density centrifugation and used directly in one-step ALP precoated ELISpot^PRO^ kits (MABTECH Inc., Mariemont, OH) for the detection of monkey IFN-γ according to the manufacturer’s protocols. Briefly, 1x10^5^ PBMC were used per well and incubated with pools of overlapping 11-mer peptides for approximately 18 hours at 37°C in 5% CO2. 7 peptide pools represented the amino acid sequences of H1N1pdm HA; peptides were obtained from BEI Resources (Manassas, VA). Peptides in pools were diluted to a final concentration of 1 mM each. Each plate contained a negative (no peptide) and positive (concanavalin A) control. Results are expressed as the average number of spot-forming cells (SFC) per 10^5^ PBMC detected for each pool, with the background (average number of SFC/10^5^ in negative control wells) subtracted. Experiments were conducted in duplicate and results were considered positive if wells contained an average of ≥5 spots and the average SFC/10^5^ detected in peptide-containing wells was at least threefold higher than the average number of spots in negative control wells.

### Statistical analysis

In order to account for multiple testing (across time points and sample sites) when testing for treatment differences in viral titers, we used a permutation test approach inspired by a method in Burrage et al. 2010 [37]. Consider the data as a matrix with rows being individual macaques and columns being treatment assignment followed by responses at different time points in the two sample sites (BAL and nasal wash; note we omitted the nasal wash titers at 10 days post infection and BAL at 7 days post infection, because there was no variability in the titers at these time points. For each of 10,000 permutation replicates, we permuted (i.e., shuffled) the treatment assignments relative to the responses (preserving the associations among responses at different times in the two sample sites) and applied the Kruskal-Wallis test for each response column. This gives a set of test statistics, *X*_*ij*_, for permutation replicate *i* with response column *j*. To control the family-wise error rate (FWER) we first calculated the ranks of the statistics for each column, *R*_*ij*_, and calculated the maximum rank for each permutation replicate across columns, *M*_*i*_ = max _*j*_ *R*_*ij*_ [38]. The 95^th^ percentile of the *M*_*i*_ can be treated as a 5% significance threshold. We consider the observed Kruskal-Wallis test statistics, for the observed data without permutation, find the corresponding rank (by comparing the observed statistic to the *Ri*_*j*_ for the corresponding response column), and then either compare it to the 95^th^ percentile of the *M*_*i*_ (for a test with alpha = 0.05), or calculate a P-value as the proportion of the *M*_*i*_ that are greater or equal to that rank.

A similar approach was applied in order to assess statistical significance in the pairwise comparisons among the three treatment groups. We focused only on BAL at 4 days post infection, because that was the only site/time for which we had evidence of a vaccine effect. We again permuted the treatment assignments relative to the response, calculated Kruskal-Wallis test statistics for each of the pairwise comparisons, and repeated the process for 10,000 permutation replicates. We then converted the statistics to ranks and for each permutation replicate calculated the maximum rank across the three comparisons. For the observed test statistics, for the observed data without permutation, we found the corresponding ranks and then calculated P-values as the proportion of the maximum ranks that were greater or equal to what was observed.

Linear regression of log-transformed viral titers on ADCC antibodies was performed using GraphPad Prism version 6.

### Data availability statement

Raw data used to generate tables and figures in this proposal are presented in S1 Appendix.

## Results

### Partial heterosubtypic protection by MVA expressing mosaic H5 protein

Rhesus macaques in this study were first vaccinated with either MVA encoding the mosaic H5 protein (MVA-H5M; n = 7; 1x10^8^ pfu/dose delivered intradermally) or a DNA vaccine encoding the same protein (pCMV-H5M; n = 6; 16 μg/dose delivered transdermally). 14 days later, all animals received a booster dose (1x10^8^ pfu) of MVA-H5M. We challenged these 13 animals, together with 2 naïve non-vaccinated control animals, with 9x10^6^ pfu of H1N1pdm A/California/04/2009. 6 naïve rhesus macaques previously challenged with the same dose and stock of A/California/04/2009 (H1N1pdm) by the same route [29] were included as historical controls for viral loads.

**Fig 1:**
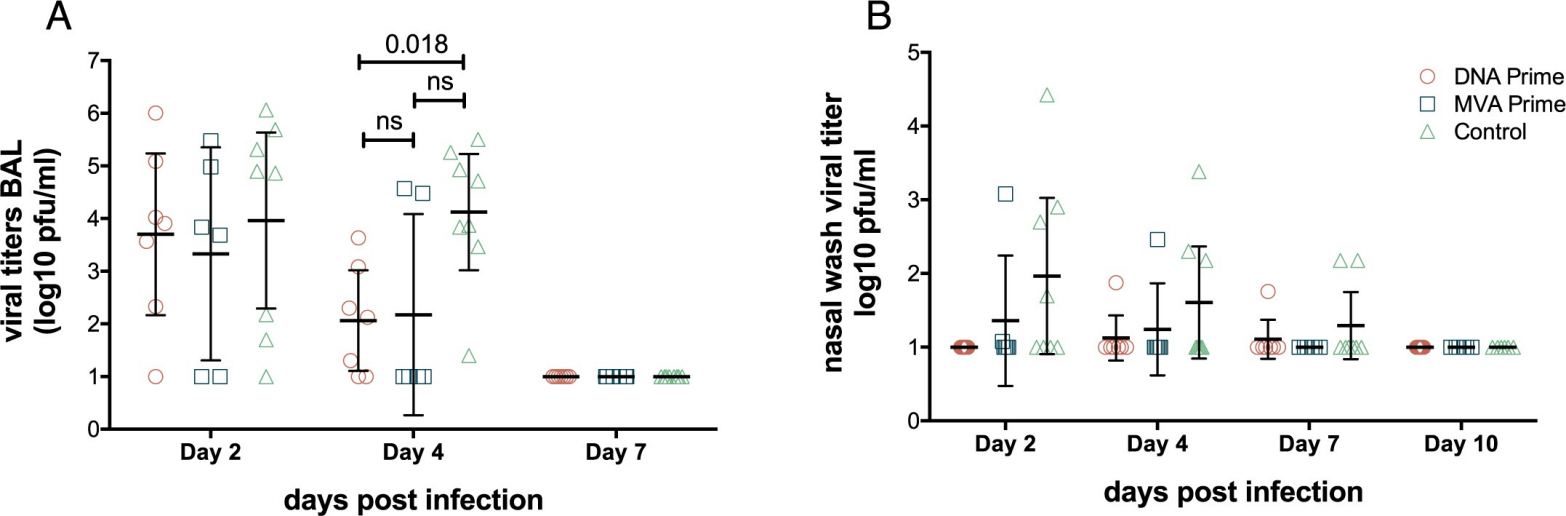
Early viral clearance from lower respiratory tract in monkeys vaccinated with an H5 mosaic MVA vaccine. Macaques were given a mosaic H5 HA antigen expressed on either plasmid DNA or in MVA and then boosted with MVA-H5M. All animals were challenged with the heterosubtypic virus A/California/04/2009 (H1N1pdm). Viral titers in (**A**) bronchoalveolar lavage (BAL) and nasal wash (**B**) were determined by standard plaque assays on MDCK cells. Control group includes 6 additional historical controls we previously published that were challenged with the same dose of the same viral stock by the same route [29]. The 2 control animals inoculated during this study are indicated by a star symbol. At day 4 post infection there was a statistically significant difference between DNA prime vaccinated and control animals as determined by a Kruskal-Wallis test combined with permutation. Bars indicate geometric mean and error bars indicate the 95% confidence interval of the geometric mean.

At frequent time points after challenge, we collected nasal washes to sample virus in the upper respiratory tract and performed bronchoalveolar lavage (BAL) to assess virus replication in the lower respiratory tract. By 2 days post infection the geometric mean viral titer in the lower respiratory tract was 3.7 log_10_pfu/ml for pCMV-H5M/MVA-H5M, 3.3 log_10_pfu/ml for MVA-H5M/MVA-H5M, and 4.0 log_10_pfu/ml for control animals, consistent with our previous studies (Fig 1A) [19,29,35]. By 4 days post-infection viral titers in BAL were significantly lower in animals vaccinated with DNA prime animals (geometric mean 2.1 log_10_pfu/ml) than in control animals (geometric mean = 4.1 pfu/ml; Kruskal-Wallis test; *p* = 0.018). Animals receiving MVA prime were not significantly different from the control animals with a geometric mean titer of 2.2 log_10_pfu/ml. By 7 days post infection, all animals had undetectable viral titers in the lower respiratory tract. In our previous study H1N1pdm replicated robustly in the lower respiratory tract, but not in the upper respiratory tract. Therefore we did not expect high viral titers in nasal wash [21]. Accordingly, animals shed infectious virus in nasal wash sporadically through day 7 post-infection, but we observed no significant differences between groups in viral titers in the upper respiratory tract. Overall, these data show that monkeys vaccinated with the H5 HA mosaic construct in a heterologous prime-boost regimen cleared the heterosubtypic influenza virus infection from the lower respiratory tract more rapidly than non-vaccinated control animals. While we did not observe a significant difference in viral loads between control animals and animals receiving a homologous MVA-H5M prime-boost, there was a visible reduction in viral loads.

The animals were also monitored for physical responses to vaccination and infection. During the study there were no significant changes in the weight of the animals. After challenge, 6 animals from the vaccine groups demonstrated abnormal coughing and sneezing. Of these, 2 animals also showed rhinorrhea, and another 2 animals had slight epistaxis. One of these animals was lethargic, and exhibited pale skin and watery eyes. However, due to the small number of animals in the study and rarity of the symptoms we observed we were unable to determine whether vaccination impacted the frequency or severity of clinical signs.

### Vaccination with mosaic HA elicits cross-reactive, non-neutralizing antibodies

**Fig 2:**
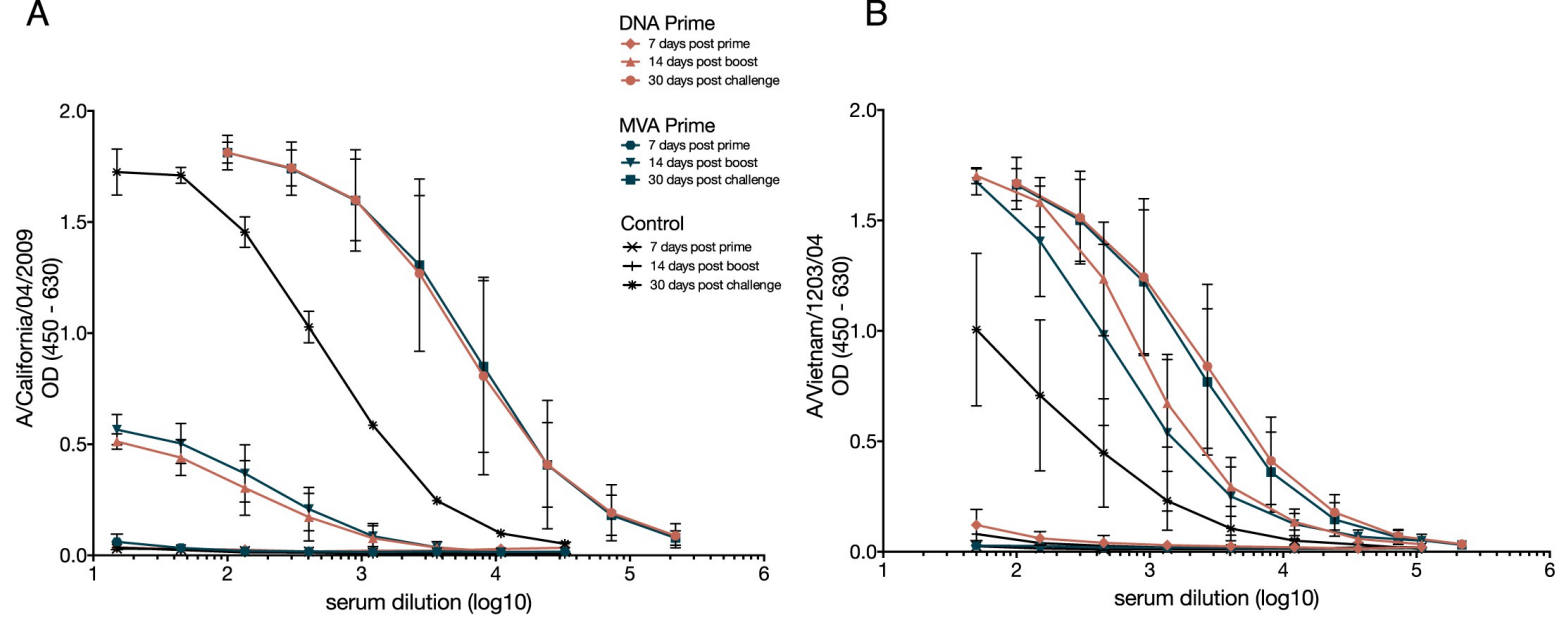
The H5 mosaic MVA vaccine stimulates antibodies against both H1N1 and H5N1 HA subtypes after prime and boost. Antibody binding to HA was measured using enzyme-linked immunosorbent assay (ELISA). Purified HA protein from A/California/04/2009 (H1N1pdm) (**A**) or A/Vietnam/1203/2004 (H5N1) (**B**) was used to capture HA antibodies in an ELISA assay. Symbols indicate mean OD; error bars indicate standard deviation.

We next asked if early clearance of H1N1pdm challenge in vaccinated animals was associated with HA-specific antibodies. Using an enzyme-linked immunosorbent assay (ELISA) we measured plasma antibodies specific for recombinant HA proteins from H1N1pdm A/California/04/2009 and the H5N1 virus A/Vietnam/1203/2004. Antibodies capable of binding HA proteins from both H1N1pdm and H5N1 were detectable 14 days after MVA-H5M boost and 30 days after challenge (Fig 2). These antibodies were not present before vaccination and were present through the day of challenge (Panels A and B in S1 Fig). There were lower levels of plasma antibody capable of binding H1N1pdm HA compared to H5N1 HA after boost (Fig 2). We observed a slight increase in H5N1 HA-binding antibodies from 14 days post-boost to 30 days after H1N1 challenge in animals primed with MVA-H5M (Fig 2B). Overall, these data suggest that vaccination generated cross-reactive antibodies capable of recognizing both H5N1 and H1N1pdm, and that H1N1pdm infection may also boost antibodies that bind to H5N1 HA.

**Fig 3:**
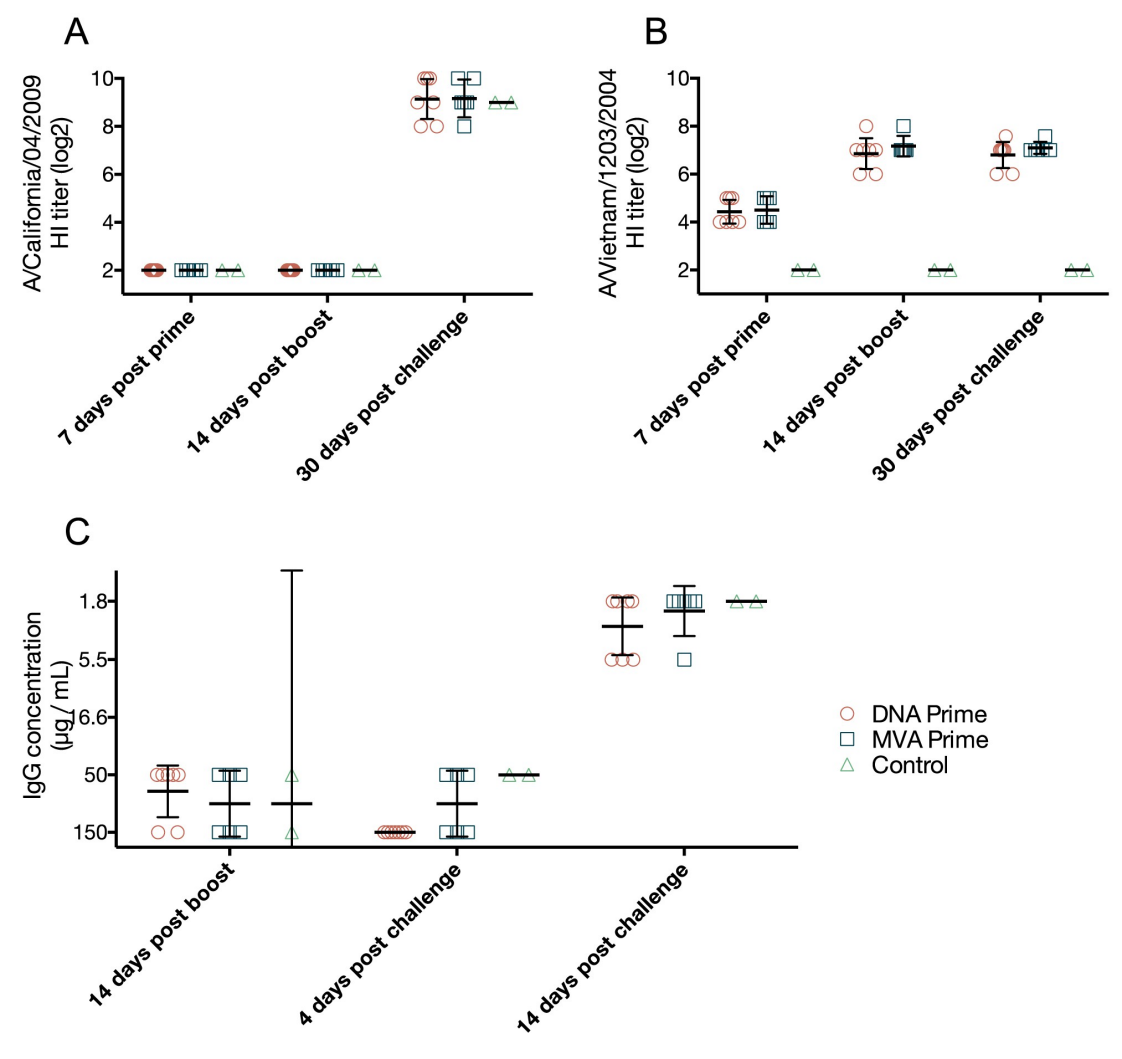
Vaccination with mosaic H5 MVA stimulates neutralizing and HI antibodies against H5N1 only. HI antibody titer was determined using a standard HI assay against both H1N1pdm A/California/04/2009 and H5N1 A/Vietnam/1203/2004. HI antibodies against H1N1pdm were undetectable until 30 days after challenge (**A**). HI antibodies against H5N1 were detected as early as 7 days after prime (**B**). Neutralization against the challenge H1N1pdm A/California/04/2009 virus was measured using plaque reduction neutralization test, where neutralization was only detected 14 days post challenge (**C**). Data points represent individual monkeys and report the IgG concentration where a 50% reduction in plaque formation is observed, bars indicate geometric mean and error bars indicated the 95% confidence interval of the geometric mean.

We next evaluated antibody responses using hemagglutination inhibition (HI), a proxy for neutralization that is commonly used to assess vaccine immunogenicity, and the plaque reduction neutralization test (PRNT), which directly tests whether plasma antibody inhibits productive viral infection. There were no HI antibodies specific for H1N1pdm until 30 days after challenge (Fig 3A). Similarly, we were unable to detect antibodies capable of neutralizing H1N1pdm in the PRNT in any animals until 14 days post-challenge, at which time such antibodies were detectable in both vaccinated and naïve animals (Fig 3C and panel C in S1 Fig). HI antibodies against H5N1 were detected as early as 7 days post-prime, but were not present before vaccination (Fig 3B and panel D in S1 Fig). At day 7 post-prime, we detected HI antibodies against H5N1, while ELISA assays did not detect strong HA-binding responses. We speculate that, at this early timepoint, IgM was the predominant antibody class. Neutralizing IgM would be detectable in HI assays, but not by IgG-specific ELISA. Overall, these findings are consistent with our previous observations in rhesus macaques vaccinated with MVA encoding HA from the H5N1 virus A/Vietnam/1203/2004 [19].

### Vaccine-induced ADCC antibodies and their association with early viral clearance

Non-neutralizing antibodies can perform effector functions, like activating complement or natural killer cells (NK cells), that assist in clearance of viral pathogens. Antibody bound to cell-surface antigens can activate NK cells to kill the antibody-bound target cell in antibody-dependent cell-mediated cytotoxicity (ADCC). As a proxy for antibody-dependent, antigen-specific NK cell killing activity, we evaluated the ability of plasma antibodies elicited by MVA-H5M vaccination to stimulate NK cell degranulation in the presence of recombinant HA, in a modification of a method we previously published [19,35]. Briefly, HA proteins are immobilized on a 96-well plate and then incubated with plasma from vaccinated or infected monkeys. Cells of the NK cell line KHYG-1 modified to express CD16 [36] are then added as effectors. Engagement of CD16 by antibody bound to the immobilized HA proteins activates the KHYG-1 cells, causing them to release cytotoxic granules. To assess the concentration of ADCC antibodies, we measured the frequency of KHYG-1 cells expressing CD107a, a marker for recent degranulation, in the presence of HA antigen and macaque plasma.

**Fig 4:**
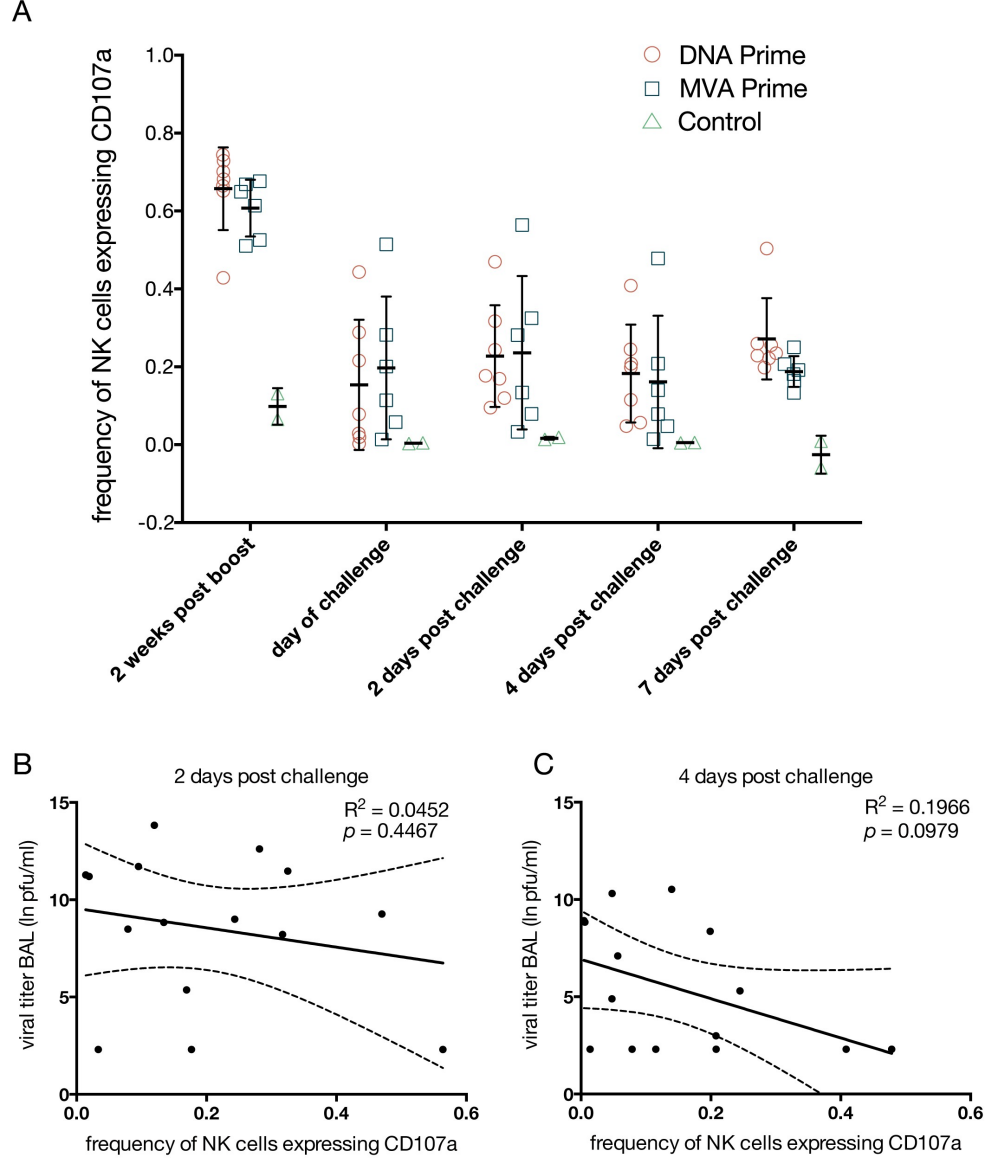
Mosaic H5 MVA vaccination stimulates antibodies capable of antibody dependent cell mediated cytotoxicity (ADCC), which are maintained throughout infection. We determined ADCC antibody titer against the H1N1pdm A/California/04/2009 using CD107a to detect degranulation. ADCC antibodies were detected at high magnitude after the MVA boost and were then maintained at a lower level throughout challenge (**A**). Data points indicate individual monkeys with the bar and error bar’s indicating median and standard deviation. Regressing viral titers on ADCC antibodies at 2 days post challenge shows only 5% of the variability in viral titer can be explained by ADCC antibodies and the association is not significant, R^2^ = 0.0452, *p* = 0.4467 (**B**). At 4 days post challenge 20% of the variability in viral titer can be explained by ADCC antibodies and the association is not significant, R^2^ = 0.1966, *p* = 0.0979 (**C**). The dashed line represents the 95% confidence interval of the linear regression model.

At 14 days post boost, a large percentage of NK cells degranulated in the presence of plasma and the A/California/04/2009 (H1N1pdm) HA, ranging from 42% to 74% of NK cells (Fig 4A). On the day of challenge there were fewer ADCC antibodies, with surface CD107a expressed on 0% to 56% of NK cells (Fig 4A). H1N1pdm-specific ADCC antibodies remained at this lower level through the rest of infection (Fig 4A). These data show the MVA-H5M vaccine was able to elicit high levels of ADCC antibodies after boost, which fell to a lower level that was maintained through infection. There was no significant difference in the level of ADCC antibodies, as indicated by the frequency of CD107a-expressing NK cells, between recipients of the DNA or MVA primes. There was no correlation between viral titers and the frequency of degranulating NK cells in the ADCC assay at 2 days post challenge; R^2^ = 0.0452, *p* = 0.4467 (Fig 4B). At 4 days post challenge there was a trend toward a weak inverse correlation between viral titers and ADCC antibodies, although this did not reach significance: R^2^ = 0.1966, *p* = 0.0979 (Fig 4C).

### Limited role for T-cells in viral clearance

**Fig 5:**
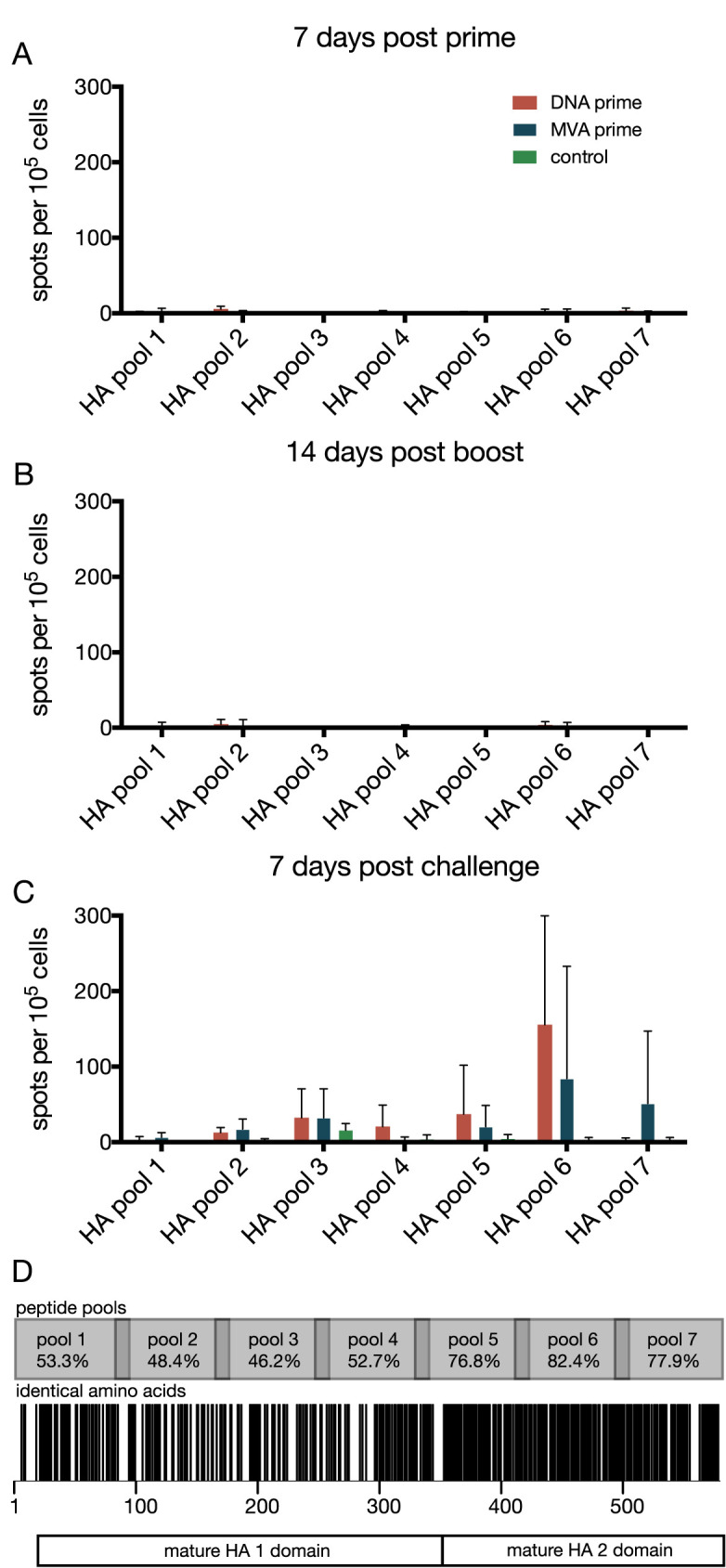
Animals mount T cell responses against the H1N1pdm challenge after vaccination with H5 mosaic MVA vaccine. We detected T cell responses in peripheral blood mononuclear cells (PBMC) using an IFN-γ elispot assay. PBMCs were stimulated with 7 pools of overlapping synthetic peptides from HA. We detected no responses 7 days after prime (**A**) or 14 days after boost (**B**). T cell responses were detected 7 days after challenge, with most responses detected in PBMCs stimulated with peptide pool 6 (**C**). The H5 mosaic HA used in vaccination had the most amino acid sequence identity shared with the challenge strain A/California/04/2009 (H1N1pdm) in the HA 2 domain, with the highest sequence identity of 82.4% occurring within peptide pool 6 (identical amino acid residues indicated by a black stripe; **D**). Error bars in A, B, and C indicate standard deviation.

Cytotoxic CD8+ T cells can also play a critical role in cross-reactive immunity against influenza viruses in animal models, including macaques [29,39]. To determine if T cell responses to vaccination and challenge might be involved in faster viral clearance, we used interferon (IFN)-γ Elispot to determine whether vaccination and/or challenge stimulated T cells capable of recognizing HA from the challenge virus. We stimulated PBMC using 7 pools of 15- to 16-mer peptides overlapping by 11 amino acids, representing A/California/04/2009 (H1N1pdm) HA (Fig 5). Due to limited sample availability, we focused on peptides representing H1N1pdm because we aimed to detect T cells capable of recognizing the challenge virus; cells recognizing the vaccine, but not the challenge virus would not likely be involved in protection. The HA protein of CA04 (H1N1pdm) and the H5 mosaic HA are 62.6% amino acid identical, with most of the shared amino acids occurring within the HA2 domain (Fig 5D).

There was little to no IFN-γ expression in PBMC stimulated with HA peptides after either the prime or boost vaccinations (Fig 5A and 5B). However, we detected low-frequency HA-specific T cell responses in vaccinated animals 7 days after challenge, a time at which T cell responses were barely detectable in non-vaccinated control animals (Fig 5C). HA peptide pool 6 elicited the highest frequency of IFN-γ secreting cells (mean = 155 spots/10^5^ cells pCMV-H5M prime, 84 spots/10^5^ cells MVA-H5M prime) (Fig 5C). Of the HA peptide pools, pool 6 has the highest amino acid sequence identity (82.4%) between H1N1pdm and the mosaic H5 HA (Fig 5D). These data suggest that, although T cell responses to vaccination were weak, MVA-vectored vaccines may have primed cross-reactive T cells that were recalled upon challenge with the heterosubtypic H1N1 virus, and may therefore have been involved in virus clearance.

## Discussion

The constant antigenic evolution of influenza viruses and its public health consequences has spurred efforts to design “universal” vaccines that could provide protection against multiple, or even all, influenza virus strains [14,40–44]. In this study we found that a mosaic H5 HA protein expressed by a modified vaccinia Ankara vector elicited cross-reactive responses against H1N1pdm in rhesus macaques and facilitated early clearance of a heterosubtypic H1N1pdm isolate.

Effective protection against influenza viruses is associated with neutralizing antibodies against the globular head of HA [8,45,46]. Consistent with our previous findings using H5 vaccines, we detected non-neutralizing antibodies capable of binding H1N1pdm HA before challenge in vaccinated macaques (Figs 2A & 3) [19]. In our previous study we reasoned that these binding, non-neutralizing antibodies might promote viral clearance via antibody-dependent cell-mediated cytotoxicity (ADCC). In the present work, vaccination with MVA-H5M elicited high levels of H1N1pdm-specific ADCC antibodies that were then maintained at a lower level through infection (Fig 4A). This is consistent with the premise that IgG1 and IgG3 antibodies, which are known to bind FcRγIII (CD16) receptors on natural killer cells stimulating degranulation, have average half-lives *in vivo* of 21 and 7.1 days respectively [47,48]. Any ADCC antibodies present at the time of challenge are most likely either remaining IgG1 antibodies or are produced by long-lived plasmablasts [49–51]. These findings should be taken into account in further studies to better understand the dynamics of ADCC antibodies after vaccination.

Currently used inactivated and live attenuated influenza vaccines can provide modest boosts to ADCC antibody responses in humans [52]. However, the trivalent inactivated influenza vaccine did not elicit ADCC antibodies in naive pigtail macaques [53], suggesting that inactivated vaccines can boost pre-existing ADCC antibodies, but do not effectively stimulate such responses de novo. Here and in our previous study we have shown the MVA vector combined with an H5 antigen is capable of eliciting a de-novo ADCC antibody response in a naïve non-human primate, raising the possibility that this modality can more effectively stimulate ADCC antibodies than standard inactivated vaccines can [19].

The MVA vector has been used to stimulate influenza-virus-specific CD4^+^ and CD8^+^ T cell immune responses, which can target epitopes that are highly conserved among influenza virus strains and subtypes [54–56]. Our previous study of H5 vaccines in non-human primates reported few virus-specific CD4^+^ and CD8^+^ T cell responses using intracellular cytokine staining [19]. In contrast, in the current study IFN-γ Elispot detected a modest T cell response in vaccinated, but not control, animals at 7 days after challenge (Fig 5C). Peptide pool 6 contained peptides that were highly conserved between H1N1pdm challenge and the mosaic H5 (Fig 5D). This suggests that the mosaic H5 vaccine stimulated a small population of cross-reactive memory T cells that was undetectable during the vaccine phase, but expanded after heterosubtypic challenge.

MVA is an extremely safe vaccine modality that was administered as a primary smallpox vaccine to over 120,000 people without adverse effects [57]. Due to this safety profile, there has been longstanding interest in using MVA as a vector for recombinant vaccines. MVA-vectored vaccines can elicit strong antibody and T cell responses [54,58]. In our study MVA-H5M elicited HI antibodies against the homologous H5 HA antigen at titers similar to those reported in human recipients in an MVA H5N1 clinical trial [58]. While we did not observe a T cell response to HA after vaccination, we did detect cross-reactive antibodies that were able to stimulate ADCC responses. MVA-vectored vaccines have elicited T cell responses in humans, but ADCC-antibodies have not been examined [54,59]. Similar to our previous MVA study in non-human primates, the virus was cleared from the lower lung faster in vaccinated than unvaccinated animals [19]. Although technical improvements to our assay prevent us from directly comparing ADCC antibody levels in this and our previous study, results from both studies support the idea that cross-reactive ADCC antibodies can reduce the duration and potential severity of influenza virus infection.

DNA vaccines by themselves have limited immunogenicity in non-human primates, but have demonstrated the ability to prime antibody and cellular responses [39,60–62]. Previous studies have evaluated DNA vaccines against influenza virus, which encoded a combination of HA, NP, and sometimes M1 or NA [39,60,61]. While our construct included only the mosaic HA antigen, we found no significant difference between groups primed with MVA-H5M or pCMV-H5M in the magnitude or breadth of T cell responses or in viral titers after challenge. This suggests that our heterologous prime-boost strategy with DNA prime did not enhance the development of T cell responses. Instead, vaccine-induced cross-reactive antibody responses may be sufficient to limit heterosubtypic viral replication.

Here we showed that monkeys vaccinated with MVA encoding a mosaic H5 HA cleared a heterosubtypic influenza virus challenge more rapidly than naïve animals. This rapid clearance was associated with vaccine-induced cross-reactive ADCC antibodies, though we could not completely rule out a potential contribution from vaccine-primed cross-reactive T cell responses. These findings provide evidence in a translational model that ADCC antibodies can be induced by vaccination and may be involved in cross-reactive immunity to influenza viruses.

## Supporting information

S1 Fig Antibodies against H1N1 or H5N1 were not present before vaccination. Antibody binding to HA was measured using enzyme-linked immunosorbent assay (ELISA). Purified HA protein from A/California/04/2009 (H1N1pdm) (**A**) or A/Vietnam/1203/2004 (H5N1) (**B**) was used to capture HA antibodies in an ELISA assay. Symbols indicate mean OD; error bars indicate standard deviation. HI antibody titer was determined using a standard HI assay against both H1N1pdm A/California/04/2009 and H5N1 A/Vietnam/1203/2004. HI antibodies against H1N1pdm were undetectable through challenge (**C**). HI antibodies against H5N1 were detected at the time of challenge (**D**). Bars indicate geometric mean and error bars indicated the 95% confidence interval of the geometric mean.
(EPS)

S1 Appendix Raw data used in this study. Raw data values used to create the figures in this manuscript are provided in this appendix.
(XLSX)
